# Palliative care for older people – exploring the views of doctors and nurses from different fields in Germany

**DOI:** 10.1186/1472-684X-8-7

**Published:** 2009-06-23

**Authors:** Torben Brueckner, Martin Schumacher, Nils Schneider

**Affiliations:** 1 Hannover Medical School, Department of Epidemiology, Social Medicine and Health System Research, Carl-Neuberg-Str.1, D-30625 Hannover, Germany

## Abstract

**Background:**

Providing appropriate palliative care for older people is a major task for health care systems worldwide, and up to now it has also been one of the most neglected. Focusing on the German health care system, we sought to explore the attitudes of health professionals regarding their understanding of palliative care for older patients and its implementation.

**Methods:**

In a qualitative study design, focus groups were established consisting of general practitioners, geriatricians, palliative care physicians, palliative care nurses and general nurses (a total of 29 participants). The group discussions were recorded, transcribed, coded and analysed using the methodological approach of Qualitative Description.

**Results:**

Deficiencies in teamwork and conflicting role definitions between doctors and nurses and between family practitioners and medical specialists were found to be central problems affecting the provision of appropriate palliative care for older people. It was emphasized that there are great advantages to family doctors playing a leading role, as they usually have the longest contacts to the patients. However, the professional qualifications of family doctors were to some extent criticized. The general practitioners for their part criticized the increasing specialization on the field of palliative care. All groups complained that the German compensation system gives insufficient consideration to the time-consuming care of older patients, and about excessive bureaucracy.

**Conclusion:**

General practitioners are the central health professionals in the delivery of palliative care for older people. They should however be encouraged to involve specialized services such as palliative care teams where necessary. With the German health care reform of 2007, a legal framework has been created that allows for this. As far as its realization is concerned, it must be ensured that the spotlight remains on the needs of the patients and not on policy conflicts and rivalries between health care professionals. Older people might particularly benefit if "talking" medicine and time-consuming care were properly catered for, financially and organizationally, in the health care system.

## Background

Health care for older people is a major weak point in many countries. In Germany, for example, the *Advisory Council on the Assessment of Developments in the Health Care System *[1] in its latest report called for new forms of cooperation between health care professionals and for the implementation of professional role changes in order to ensure appropriate health care for older patients. An example of the obstacles to overcome is the traditionally pronounced centering on curative medicine combined with significant coordination problems and competition between various medical disciplines and health professionals [2].

In order to be able to implement changes it is important to clarify and analyse the attitudes and perceptions of the involved stakeholders towards different aspects of health care provision for older people as we know that attitudes and perceptions can significantly differ depending on the health professionals' specialization and major activities [3-5]. Regarding palliative care the available studies on the views of different stakeholders mainly focus on palliative care in general or on the group of cancer patients. Few is known in this context in regard to the specific target group of older people, especially for Germany where to our knowledge thus far there are no such studies available.

That is why we wanted to explore the views of doctors and nurses from different areas on palliative care for older people within the framework of the German health care system. In particular, our aim was to learn more about the health professionals' understanding of the responsibilities of providing palliative care for older people, their roles, cooperation with other health professionals and the health system-related framework.

### Setting

In Germany there is a wide-spread undersupply regarding specialist palliative care both for inpatients and in particular for outpatients. At present, in Germany there are about 330 hospices and palliative care units with a total of about 2800 beds (accordingly, about 34 beds per one million inhabitants) and nationwide a total of about 60 palliative care teams (for 80 million inhabitants). However, because of the heterogeneous structures and inconsistent definitions it is hard to state exact numbers. Additionally, there are considerable regional differences with partly very well developed services in some urban areas and extensive gaps in the periphery [6,7].

Besides, there are problematical deficits concerning the education and the advanced training of all health care professions regarding palliative care; for example, the subject of palliative medicine is not compulsory – and therefore only punctually taught – in medical schools [8]. Advanced training in palliative care is held on voluntary base, so that the majority of physicians, nurses and other health professions have insufficient palliative care competencies.

However, in recent years palliative care has increasingly been recognized in public and politics. For example, a big step in 2002 was the introduction of the voluntary option for physicians to gain the minor specialization in palliative care, and another one was in 2007 the legal introduction of specialist outpatient palliative care (SAPV) with the most recent health care reform. According to the law, specialized outpatient palliative care contains specialist medical and nursing services including the coordination of the health care delivery. The aim is to provide care to the patients in their familiar home environment. The target groups are compulsory insured patients with a non-curable, advanced and advancing disease with limited life-expectancy and an extensive demand for care. To receive SAPV at the expense of the compulsory health insurance, the patient needs to have a prescription by a specialized physician, a family doctor or a hospital doctor. So far, SAPV is only realized in some few regions in Germany. There are no uniform standards regarding the personal and structural requirements of specialized services, and their cooperation with other actors (especially family doctors and nursing services) is not consistently regulated [9].

In general, in Germany the patients can widely choose specialized physicians and family doctors without any restrictions. In 2004, a practice charge ("Praxisgebuehr") of Euro 10 for the first contact at a physician's office in every quarter of the year was introduced. As a first step towards a GP gate keeper role they have to pay an additional fee if they do not consult their GP first. Moreover, the GP can determine if the specialist is allowed to refer the patient to other medical specialists or if he has to be referred back to him after the consultation. These regulations apply to the approximately 90% of the German population which has state health insurance. The remaining 10% (mainly self-employed, civil servants and high income groups) are covered by private health insurance [10,11].

In light of these policy changes, the aim of this study was to explore various health providers' perceptions and attitudes concerning the delivery of palliative care to older persons in Germany.

## Methods

### Sampling

We performed seven focus group discussions with doctors and nurses from different fields (with a total of 29 participants). The inclusion criteria for the participants, their recruitment and their sociodemografic data are shown in [Additional file 1].

The invitation to participate took place in different ways: With respect to the geriatrics, the head of department in all specialist geriatric hospitals (N = 3) in the Hannover region were asked to participate and to forward the invitation to other physicians in the hospital. Palliative care physicians and nurses were recruited from the palliative care units, palliative care teams and hospices in Hannover (each N = 3) by personal invitations. General practitioners were invited during a meeting of academic teaching physicians in the Institute for General Medicine at Hannover Medical School. For the recruitment of the general nurses we addressed invitations to the heads of nursing homes and outpatient nursing services selected by personal contacts of the study team. For each group we asked the first professionals who agreed to participate to forward our invitation to other professionals in their institution (snowball approach). Due to this recruitment strategy the total number of professionals that were asked to participate cannot be calculated. However, we can state as a resumé that the recruitment was difficult with respect to the general practitioners, geriatricians and the general nurses and comparably unproblematic with respect to the palliative care specialists (doctors and nurses).

Every one of the first five focus groups was monoprofessional; more precisely, there were separate groups of general practitioners (Group 1, Ha), geriatricians (Group 2, Ga), palliative care physicians (Group 3, Pa), general nurses (Group 4: Pf) and palliative care nurses (Group 5, Pc). The selection of these professionals and disciplines is based on the assessment that their views are particularly meaningful for the clinical questions obtained from the literature and medical care practice. First of all, the goal was to explore the points of view of different professionals and disciplines separate from one another.

Then we performed two profession- and discipline-overlapping, mixed focus groups to further develop the subject with intense debate. The mixed focus groups 6 and 7 were exclusively composed of participants from Groups 1–5. Therefore, in the end, we asked all participants of focus groups 1–5 if they were interested in participating in a following mixed focus group. [Additional file 2] presents the characteristics of the focus groups.

The relevant individuals and institutions have been provided with written information on the background, process and objectives of the study and have been invited to participate. A consensus was reached with the interested persons on a date for the performance of the focus groups. The participants received a compensation of 50 Euros.

### Data collection

The focus groups took place in the period from August 2007 to May 2008. All group interviews were conducted at Hannover Medical School, Institute for Epidemiology, Social Medicine and Health Services Research. Discussions were moderated by MS who was assisted by TB or NSCH. The moderator largely abstained from involvement in matters of content. He was actively involved for example in matters of questions and definitions and for transitioning to the next question. Furthermore, he ensured a fair distribution of speaking time for all actors and occasionally directed questions to individual participants to include them in the group discussion.

Each group began with a round of introductions including information about age, location and practical experience of the participants [Additional file 1], followed by an introduction in methodic aspects and terminology. Regarding the term palliative care, we referred to the definition of the World Health Organization [12] in order to avoid misunderstandings and discussions.

In the course of each of the focus groups 1–5, we used a topic guide based on research questions, literature studies and our clinical experience. The topic guide consisted of central questions [Additional file 3] which were individually presented on a separate slide to structure the discussions. According to the themes emerging during the analyses of the data from groups 1–5, we further developed central questions for group no. 6.

At this point in the study, two external experts in the field of geriatric palliative care from Austria and Norway were involved (see acknowledgements). They reviewed our summary material from groups 1–5, evaluated it against the backdrop of their personal experience and provided suggestions for further questions. Our goal with the involvement of external experts was to consider broader perspectives in the interpretation and development of the focus group work.

The guide was further developed according to the aspects that came up during the analyses and on the basis of the external expertises, resulting in the central questions for focus group no.7.

### Analysis

The focus groups lasted between 90 and 130 minutes. They were digitally recorded, transcribed and evaluated for analytical content. Audio recording and transcripts were then reviewed for conformity.

The transcripts were initially reread for the first incorporation into the data material and then a systematic evaluation was performed with the software package ATLAS.ti [13]. The data analysis was based on the approach of Qualitative Description [14]. In the first step, the group discussions were outrightly coded and codes were developed from the material. In the subsequent phase of axial coding, the identified codes were summarized into categories. In the third phase of selective coding, the core categories on which the interpretation of the group discussions was constructed were then worked out.

In order to find out differences regarding the views of the health professionals' roles within the provision of palliative care for older people, we conducted comparative analyses by professional discipline with focus on general practitioners to specialists.

The data analysis was based on the study of the literature, the external expert opinions and the practical clinical and scientific experience and qualifications of those responsible for the study: TB is a medical student in the last year of clinical training with a focus on oncology and palliative medicine. MS is a psychologist and health care scientist (Public Health) and NSCH consultant in family medicine and palliative medicine as well as a health care scientist and lecturer in Public Health and health services research.

The development of analyses and questions was continually and intensively discussed among the authors to avoid misunderstandings and to consequently summarize the results. The strategy of multiple coders and analysts is recommended by Miles and Huberman in order to reduce bias and to improve the validity of the results [15].

### Ethics

After consultation with the authorities, formal approval of the study by the Ethics Committee of Hannover Medical School was not necessary because no patient data was collected and no experiments were performed on human beings. All participants were informed of the background, process and objectives of the study and were able at any time to end their participation without giving any reasons. All data were anonymously treated and exclusively used for scientific purposes.

## Results

The content analysis of the focus group transcripts resulted in four main categories consisting of several subcategories [Additional file 4]. The roles of different medical disciplines within the provision of palliative care for older people were discussed in a very intensive way so that we focus the following presentation on those results. The additional topics are presented in a shorter way.

### I Stakeholders involved in geriatric palliative care

In order to find out which stakeholders are important within the context of palliative care for older patients, the interviewees were asked to list all those people, professions, institutions and facilities that spontaneously crossed their mind. The following items were mentioned by all the interview groups: *family members, volunteers, nurses and general practitioners*. Except for the group of palliative care physicians, all other groups also named the term "physicians" in general. However, the terms *palliative care physician, palliative care nurses*, and *palliative care teams *were only named twice and the term *geriatrician *only once.

#### Roles of different medical disciplines

The discussion of the roles of the different medical disciplines within the context of palliative care for older people was very intense and controversial.

#### Opinions of general practitioners

The general practitioners said that family doctors as well as palliative care physicians and geriatricians argued for a holistic approach to patient care. However, the general practitioners stated that they were the central contact person for the patients and therefore had the most comprehensive approach whereas specialists were only partially involved.

*"Family medicine is at the top. Geriatrics and palliative medicine are parts of our work. (...) there may be times that you will consult with experts in this field. But we ourselves must serve as the basis of the care." *(Ha1-3)

On the other hand general practitioners saw an imminent decoupling of palliative medicine from general medicine. They found the reason for this in the increasing specialization which was strongly introduced for the profession of anaesthesiology in Germany and which they judge to be rather harmful. To undermine their central role in palliative care, general practitioners claimed that they have always provided palliative medicine. It was just not labelled as palliative medicine:

"*Family medicine has actually always been palliative medicine. We just haven't defined it as this (...) For our older colleagues the term palliative medicine just doesn't do anything for them. (...) They say that we have always done this. What do you need with this new-fangled stuff?*" (Ha1-3)

Furthermore, general practitioners estimate themselves to have the best qualifications in the broad field of palliative care for older people as they had an extensive relationship with their patients as well as with their social environment and, therefore, had a detailed knowledge of their biography. In contrast geriatricians and palliative care physicians were involved with older patients in special cases and settings only; therefore they overviewed fragments of older patient care only.

As another argument in favour of general practitioners, cost-efficiency was pointed out: *"And I also think that the way in which palliative medicine functions is somewhat different from the way family medicine works; because general practitioners have learned in our health care system to achieve a lot with as little expenditure as possible; as cost-efficiently as possible, because it is good to work under an economic aspect." *(Ha1-2)

#### Opinions of geriatricians

The geriatricians saw the main difference between themselves and general practitioners in regard to the health care structures and standards in Germany. Presently, in the outpatient sector in Germany, there barely exists specialist geriatric medicine whereas in the inpatient area, there is a high degree of structures and standards available in geriatrics. The geriatricians critically assessed the skills of the general practitioners in the area of palliative medicine: *"There is, I believe, certainly a whole host of general practitioners who are very comprehensive (...) in supplying palliative care. However, some completely ignore it because they feel overwhelmed by the issue." *(Ga1-3)

On the other hand, the geriatricians positively evaluated the skills and capabilities of palliative care physicians: *"Especially when it comes to pain therapy, the palliative care physicians perform very sophisticated medicine, e.g. nerve blockages and such things. We need these specialists, because we of course don't do these things (...) because we lack the know-how." *(Ga1-3)

#### Opinions of palliative care physicians

The palliative care physicians saw similarities among the three medical disciplines that were involved in the study regarding the fact that all offer ....concomitant and integral medicine. More than many other disciplines, the three groups would have an insight into the psyche and the social environment of the patient. This approach would mean that professionals from palliative medicine, geriatrics and family medicine were generalists.

The palliative care physicians clearly delineated their field of activity from that of the general practitioners. The latter take care of a much larger variety of patients and usually are the first to, e.g., discover a cancer of a palliative patient:

*"Palliative medicine is a small area. That is clear. Straightforward and manageable. And family medicine (...) patients, children, grandparents and siblings, that is quite comprehensive. And probably also the patient from the beginning of the illness. The general practitioner himself would probably diagnose cancer disease." *(Pa1-2)

Consequently, compared to the general practitioners the palliative care physicians get involved in the care of older palliative patients relatively late, they get to know the patients only for a short time and also participate in the process at a point when the patient has usually already faced the disease for a long time.

#### Opinions of the general nurses and palliative care nurses

The general nurses consider the family doctors to be the primary caregivers for geriatric palliative patients: *"It all really starts with the family doctors. He's the one who's there at the beginning." *(Pf1-3)

Having this in mind, the nurses are critical of the fact that not all general practitioners are well trained and possess the special expertise. This would only come about now with new training programs for young physicians.

The palliative care nurses shared the opinion that general practitioners would also need palliative medicine and geriatric expertise because the family doctor's office would be the first point of contact for the patient. It was considered as being critical that in Germany the geriatricians are almost not available in the outpatient sector.

#### Knowledge, skills and expertise

In all focus groups, specialist expertise in pain therapy and symptom control was considered as a key precondition for good care for older incurably ill patients. The importance of multidisciplinarity and cooperation was highlighted multiple times: *"...you need, so to speak, high specialist expertise that cannot be achieved by each individual himself, but which somehow must be guaranteed to be available on the team." *(Ga1-3)

Besides the medical aspects, the importance of social expertise was emphasized, containing characteristics like empathy, respect, tolerance, healthy common sense and the ability to listen and to have patience. In this context, the significance of the biography of the patient was also stressed: *"And it is therefore helpful that one knows the family and the pre-history. The hobbies, the passions. Whether someone is religious or not." *(Ha1-2)

*"Again, this is the biography. This is often how one has lived one's life. And the whole pre-history of the life, how one circumvents the end of life." *(Pc2-1)

The talents to organize and improvise are also considered to be important skills in palliative care for older patients. The general practitioners mentioned the ability to make decisions and the need of courage to deviate from universal standards in certain situations. The palliative care physicians reaffirmed this commitment to flout standard rules. As an example, the allowance for alcohol and tobacco consumption among geriatric palliative care patients was named.

### II Target groups

#### Differences between older and younger palliative care patients

All the interviewed groups saw the multi-morbidity and the often resulting poly-pharmacotherapy as a major difference in the care of older palliative care patients in comparison to younger palliative care patients. It was stressed that the effects of drugs on the elderly are poorly studied and that there is a great risk of unforeseeable interactions and side effects. In addition, cognitive deficits among the elderly often lead to reduced compliance. The subject of the treatment choice and limitation was also discussed. It concerned the geriatricians "*that it was perhaps easier to achieve consensus with the families and overall on an interdisciplinary basis when it concerned old people where perhaps everyone understands that now a treatment limitation is necessary. And with a young person it is the case where one always hopes that maybe something can still be done." *(Ga1-1)

General practitioners stated another difference between older and younger palliative care patients in their attitudes towards life, illness and death: *"So, the old man who has finished with his life in general. And the proximity to death doesn't scare him very much in general (...), and for the young, I still naturally have other conflicts and also fear and concerns to work through here. And because there is a structure, because for the young I most likely also need more psychological expertise on the team." *(Ha1-3)

#### Gender-related differences

Among the palliative care physicians' gender-specific issues in regard to treatment desire were mentioned:*"We have to deal with women who have long spent their whole life being responsible for others, and still continuing to look after others, then maybe it is worthwhile to look critically at whether women simply do not want to just sit back. And whether or not they just do not want to be enabled any longer." *(Pa1-1)

The nurses and geriatricians found that woman in comparison to men often worry about other people and take care of the problems of others. This would mean that older female palliative care patients are more restrained, endure more pain and demand less pain medication. They built their lives on others and thought less about their own suffering and more about the concerns of relatives. In contrast, male older palliative care patients would be more able to complain and think more about their own suffering than a female patient in a comparable situation.

### III Inhibiting factors for the realization of geriatric palliative care

Two main aspects for inhibiting and supporting factors for the realization of geriatric palliative care were worked out: on one side, the framework of the German health care system and on the other hand, closely connected, the strong bureaucracy.

#### Health care system-related barriers

Insufficient financial resources in the health care system were seen as the main barrier to the implementation of good palliative care for older people: *"There must be more money in the system. Or the distribution of money must be worked out." *(Ha1-1)

The palliative care nurses complained about the separation of statutory health insurance and long term care insurance in Germany, resulting in sometimes unclear responsibilities and long-winded processes: *„So the cooperation between the different costs units sometimes does not function at all.‟ *(Pc3-1)

According to their own statements, the participants did not primarily desire higher salaries for themselves with their demands for more money; however, they wanted to improve medical care and work conditions. So, the general nurses proposed an increase in the key staff. In addition, they were – like the family practitioners themselves – of the opinion that the general practitioners' budgets were too strictly limited.

With the legal claim to specialist outpatient palliative care established with the most recent German health care reform in 2007, improvement concerning palliative care for older people was expected. However, critical statements were also apparent concerning the distribution of financial resources. Consequently, the geriatricians expressed the concern that some stakeholders want to tap new revenue sources without being able to offer adequate palliative care: *"...does the money always flow to where the really good palliative medicine is supplied?" *(Ga1-3)

*"And here one has apparently discovered that one can open up new means of income. Whether it is really the primary motivation to establish or to improve the palliative medical care?" *(Ga1-3)

In this context, the general practitioners saw the development of new specialized structures as being critical: *"Because palliative medicine is predominantly a family practitioner' task, why is there now suddenly basically additional specialist care ... or even specialists active in this field? I find this really strange." *(Ha2-3)

*"Palliative care units? I am still critical." *(Ha1-3)

#### Bureaucracy

Bureaucracy was seen as fundamentally necessary as it is a component of quality assurance. However, all interview groups complained about excessive bureaucracy which costs a lot of time that otherwise could be invested in a better way.

*"So you always have to write down what you have done, why you have done it. Thus, so that the colleague who is there at night knows what is actually going on. This is not the problem. For me the problem is the applications and forms of the insurance carrier. Where the cost absorption claims were placed, which can then be rejected, the objections were made, where the invoices were questioned, where you will spend hours of your work time." *(Ga1-3)

### IV Improvement of palliative care for older people

The focus group participants made a lot of suggestions to improve palliative care for older patients. Increased input of resources, especially more money and more staff was seen as being particularly effective.

In addition, there was a critical discussion on the implementation of nursing home physicians which are not established in Germany. This idea was introduced into the discussions (Group 6) as it has become a major subject of the political discussions in Germany and with the recent nursing care insurance reform in 2008, legal conditions for nursing homes to employ physicians were introduced.

#### More money, time, and communication

The approaches to improve palliative care for older people focused on the aspects of "more communication", "more time" and "more money", taking into account that these aspects are closely linked: If there was more money for palliative care, the health care professionals could have higher key staff and would consequently have more time to communicate with patients and families as well as with other professionals. Overall, that could lead to an improvement in patient-centred care. The claim for more money and the resulting increase of medical staff and hence the benefit of more time for communication was found in all focus groups, e.g.:

*"But communication always requires (...) a common space, a common time, a common language and a common attitude to one another. (...) Space and time are just (...) not there, because we do not have the possibilities financially, the resources to do that." *(Ga1-2)

*"But we don't have the resources for real conversations (...) We have only one nurse for 40, 60 patients, how should we have time for real conversations then?" (Pf1-4)*.

The participants agreed that the reason for the lack of money and the inadequate distribution of resources was found within health politics. Consequently, the participants demanded a new type of compensation rate for patient consultations and also for discussions with colleagues. In addition, treatment by occupational therapists and other professionals should be better rewarded.

Regarding bureaucratic work, the delegation of non-physicians' and non-nurses' work to other professionals (e.g. documentation assistants) should be intensified and technological systems, e.g. a paperless hospital, made more use of: *"(...) we have documentation assistants for this, or the technology solves the problem for us." *(Ga1-2)

#### Nursing home physicians

The participants of the group discussion had various opinions concerning the question if nursing home physicians should be regularly introduced in Germany. One geriatrician criticized that some general practitioners would not invest enough time for their patients living in nursing homes, a nursing home physician could have a much closer commitment to the nursing home and its residents.

*"As for such a nursing home physician I find that the argument is also appropriate here that there are general practitioner colleagues who actually just rush through, and don't care for the patients intensively (...) Particularly with the dementia patients, because they just don't get involved. And such general practitioners are then perhaps inferior to someone there who perhaps supports this institution with a certain commitment and actually also can assess the caregivers and can also actually cooperate well with these." *(Ga1-1)

According to the family doctors' opinion, another advantage is that the nursing home physician-model may improve team building with the nurses.

Also from the perspective of the nurses, a nursing home physician could be useful due to his good accessibility and because they don't have to deal with many different general practitioners: *"Every nurse wants to have a physician in the home because it would also be easier because of the logistics. So that's what the nursing staff wants. With orders and prescriptions and perhaps weekly office hours one could have a regular contact person." *(Pf1-3)

The answer of the palliative care physicians on the question which medical specialty would be most suitable to act as nursing home physician was that it should be either a family doctor or a geriatrician because of their experiences with elderly people:

*"(...) either a family doctor or a geriatrician. Because I think that they have the most experience with elderly people. And their problems." *(Pa1-1)

However, the "nursing home physician-model" was controversially discussed in principle. A general practitioner spontaneously insisted that it is contrary to the right to free choice of a doctor; she also pointed out that one problem would be that the personality of one particular nursing home physician does not always suit each patient:

*"And I know that there are many completely different types of doctors and many different types of patients. A nursing home physician cannot make all patients happy. That is just natural. It starts with gender, whether it is a man or woman." *(Ha2-3)

Likewise, it was seen as being negative that a doctor only working in a nursing home has not already been caring for the patient for years before his admission to the nursing home. Therefore, a nursing home physician would not be a part of the patient's social environment and would not know the patient's life story within the context of treatment. Patients could find it difficult to get accustomed to a new doctor as the main contact person:

*"But it is detrimental (...) the last thing that connects the elderly patient to his home is the general practitioner. Sometimes it's a piece of furniture. And at the height of dementia, the general practitioner who has for years been beside him in healthy and in sick times is the one who can quiet him and influence him." *(Ha1-3)

## Discussion

Demographic change has made palliative care for older people a highly important topic. In this study, the views held on this subject by physicians and nurses from different fields were systematically examined – as far as we know, for the first time in Germany.

The main findings highlight the fact that the central health professionals involved in delivering palliative care to older people are general practitioners, who need to be encouraged to involve specialized services such as palliative care teams where necessary. However, significant conflicts were apparent with regard to the responsibilities of different health care providers (in particular generalists vs. specialists).

### Strengths and weaknesses of the study

We chose a qualitative approach because very little is known about the attitudes of health care providers in Germany towards palliative care for older people. Focus groups are an established qualitative research method for collecting information from particular groups, e.g. health care professionals. Using this approach rather than individual interviews enabled the researchers both to explore disagreements between the participants more thoroughly and also to identify points of consensus [16-18].

At first glance, the number of participants in some of our focus groups appears low. However, we believe this to be methodologically justifiable: the small groups were quite homogenous in terms of their professional background; in the smaller groups with few participants discussions were very intense, and ultimately it proved possible to obtain as much evaluable data from these focus groups as from those with larger numbers of participants. It should also be remembered that there are still only a small number of professionals designated as specialists in palliative medicine and palliative care in Germany who fulfil our inclusion criteria, as palliative care in Germany is still a very underdeveloped field as compared to (e.g.) Great Britain [6].

Furthermore, it should be considered that it was difficult to schedule group discussions bringing together a number of physicians and nurses heavily involved in clinical work. The participants were often engaged in shift work or had to do overtime, while participating in the focus group discussions in their spare time.

Further studies on the topic should also include the points of view of other health professionals (e.g. social workers) and, in particular, the patients' and relatives' perspectives.

### Interdisciplinary rivalry

According to the literature, while general practitioners demonstrate a high level of motivation for palliative care [19], their skills do not always match up to expectations [20,21]. They see it as part of their role to be the central professional contact person for their patients at the end of life, and do not want to involve medical specialists unless it is unavoidable [22,23]. The views of the general practitioners who participated in our focus groups are consistent with this. Other participants evaluated the role of the general practitioner similarly; but the palliative care specialists in particular would have liked the general practitioners to be better qualified in palliative medicine.

Good cooperation between general practitioners and palliative care specialists could enhance competence and mutual acceptance. At present, however, sustainable structural preconditions for realizing this are still lacking in many regions of Germany [6] even though the new specialist outpatient palliative care (SAPV) introduced by the latest German health care reform in 2007 has been put into place. SAPV explicitly envisages cooperation between the new services and existing structures, especially the care delivered by family doctors, the coordination of medical care and the improvement of teamwork as between professionals [9]. It thus represents an important step in the right direction of improving palliative care for younger and older patients with and without cancer.

However, it is still unclear how specialist outpatient palliative care will be concretely provided to patients. Experts recommend a nation-wide network of multidisciplinary palliative care teams. Studies have shown that palliative care teams can reduce the numbers of days patients spend in hospital and the use of emergency services; furthermore, we know that they enhance patients' quality of life [24-26]. These results, obtained primarily with oncological patients, have also been confirmed for patients with non-malignant diseases such as chronic heart failure or COPD [27] – typical clinical pictures of geriatric patients.

However, the increasing number of people newly specializing in the field of palliative medicine was also criticized during our focus group discussions. Conflicts concerning the responsibilities of different health care providers (in particular generalists vs. specialists in palliative care) were apparent. For example, family doctors questioned the motives of other professional groups for specializing in palliative medicine (e.g. anaesthesiologists who originally had a field of activity in the hospital with little "talking medicine", but who now specialize in palliative care as "talking professionals" with outpatient focus).

In this context, the distribution of the financial resources for palliative care was seen as a critical issue. General practitioners and geriatricians pointed out that new institutions and services could arise to exploit a lucrative new area of business in palliative care.

These findings are not surprising. Interdisciplinary rivalry and strong lobbyism are well-known in other fields of the German health care system, and it seems that they have increasingly invaded palliative care now that it has become a new specialty requiring appropriate funding from the statutory health insurance system [9].

### Teamwork between doctors and nurses

It is usual in Germany for many different family doctors to provide medical care within a single nursing home. They visit their patients on demand or routinely without formal cooperation agreements or contracts with the nursing home. Most of their work is performed in their surgeries outside the nursing home. As a possible approach to improving health care for older people living in nursing homes, the establishment of a system of physicians with fixed contracts with nursing homes (so-called nursing home physicians) is under discussion. We therefore introduced this topic into the focus group discussions.

The nurses in particular viewed the idea positively, as they thought a fixed nursing home physician would lead to improved teamwork with the nurses. In addition, such specialized physicians would probably be better qualified and have more time for the medical care of elderly patients. Against this, the family doctors brought up the negative argument that nursing home physicians, in contrast to family doctors, only get to know their patients when they are admitted to the nursing home, and consequently do not learn enough about the life stories and social environments of their patients.

This negative attitude on the part of some physicians found in our study is confirmed by recent official statements, e.g. by the German Medical Association, in which it is argued that nursing home physicians would not solve the main problem of inappropriately low financing of the time-consuming medical care for nursing home residents [28]. Although the recent nursing care insurance reform of 2008 laid down the legal basis for nursing homes to employ physicians, it seems doubtful whether this approach will indeed be widely adopted. At any rate, the spectrum of possible improvements to medical care in nursing homes has been extended, and now encompasses everything from informal cooperation with family doctors and specialists to the employment of nursing home physicians.

### Lack of time

The extent to which the importance of biography work was highlighted in the group discussions is very striking. This supports, for example, the work of Norberg [29], who stressed the importance of biography work done by people nursing patients with dementia. In particular, general practitioners who have had long-term care of patients know their biographies well and are able to exploit this knowledge in palliative care when they grow older. Crooks and Agarwal [30] point out the importance of continuity of care within family practice. Continuity involves not just the medical history but the entire individual biography of the patient. For example, the elderly patient in palliative care who experienced war and the subsequent time of hardship is more acquiescent and may be reluctant to complain about his symptoms. Crooks and Agarwal suggest that general practitioners should share their specific knowledge with other health professionals in order to improve patient-centred care. However, the fact that biography work is very time-consuming, and has no place in the German health care funding system, is likely to be a barrier to this in everyday practice.

There was considerable discontent among all participants of the focus groups with the amount of time available for "talking" care in daily routine work. Reasons for this lack of time to spend with patients were seen in increasing administrative work (bureaucracy) and the low number of staff.

## Conclusion

It is reasonable that general practitioners should be the central health professionals engaged in providing palliative care for older people. They should however be encouraged to involve specialized services such as palliative care teams where necessary. With the German health care reform of 2007, a legal framework has been created that allows for this. As far as its implementation is concerned, it must be ensured that the spotlight remains on the needs of the patient and not on policy conflicts and rivalries between health care professionals.

New concepts of care such as the establishment of nursing home physicians may contribute to better palliative care for older patients. However, there appears to be room for improvement with regard to the degree of openness displayed by doctors and their representatives. Furthermore, if palliative care for older people is to be improved, barriers such as inadequate funding and the inappropriate distribution of the available resources need to be overcome, in favour of "talking" medicine and medical care.

## Competing interests

The authors declare that they have no competing interests.

## Authors' contributions

TB and MS recruited the participants and conducted and analysed the focus groups. TB drafted the manuscript. NSCH conceived the study, helped to realize and analyze the focus groups and made contributions to the manuscript. All authors read and approved the final manuscript.

## Pre-publication history

The pre-publication history for this paper can be accessed here:



## Supplementary Material

Additional file 1 **Inclusion and characteristics of the participants**. The data provided show the inclusion criteria and the demographics of the participants for each professional group. Click here for file

Additional file 2 **Focus groups**. The table presents the characteristics of the five monoprofessional and two mixed focus groups. Click here for file

Additional file 3 **Central questions for the focus group discussions**. The central questions that were posed during the focus group discussions are described. Click here for file

Additional file 4 **Main categories and subcategories**. This table shows the four main categories and the subcategories that resulted from the content analysis of the focus group transcripts. Click here for file

## References

[B1] Advisory Council on the Assessment of Developments in the Health Care System (2007). Cooperation and responsibility. Prerequisites for target-oriented health care. http://www.svr-gesundheit.de/Gutachten/Gutacht07/KF2007-engl.pdf.

[B2] Schneider N (2006). Health care in seniority: crucial questions and challenges from the perspective of health services research. Z Gerontol Geriat.

[B3] Abholz HH (2004). Generalist vs. Spezialist – Konzeptionelle Überlegungen und Ableitungen zur Versorgungsstruktur. J Public Health.

[B4] Vejlgaard T, Addington-Hall J (2005). Attitudes of Danish doctors and nurses to palliative and terminal care. Palliat Med.

[B5] Schneider N, Ebeling H, Amelung VE, Buser K (2006). Hospital doctors' attitudes towards palliative care in Germany. Palliat Med.

[B6] Schindler T (2006). Palliative care in Germany [in German]. Bundesgesundheitsblatt-Gesundheitsforschung-Gesundheitsschutz.

[B7] German Association for Palliative Medicine (2008). Palliativmedizin & Hospizarbeit 2008 (Realität & Bedarf in Deutschland). http://www.dgpalliativmedizin.de.

[B8] Schneider N, Schwartz FW (2006). High need for improvement and many open questions in health care for palliative patients [in German]. Medizinische Klinik.

[B9] Schneider N (2008). New specialist outpatient palliative care – a position paper [in German]. Z Allg Med.

[B10] Rückert IM, Boecken J, Mielck A (2008). Are German patients burdened by the practice charge for physician visits ('Praxisgebuehr')? A cross sectional analysis of socio-economic and health related factors. BMC Health Serv Res.

[B11] Rosemann T, Joest K, Körner T, Schaefert R, Heiderhoff M, Szecsenyi J (2006). How can the practice nurse be more involved in the care of the chronically ill? The perspectives of GPs, patients and practice nurses. BMC Fam Pract.

[B12] Sepulveda C, Marlin A, Yoshida T, Ullrich A (2002). Palliative care: The World Health Organization*'s *Global Perspective. J Pain Symptom Manage.

[B13] Scientific Software Development (1997). ATLAS.ti – the Knowledge Workbench. http://www.atlasti.com.

[B14] Milne J, Oberle K (2005). Enhancing rigor in qualitative description: a case study. J Wound Ostomy Continence Nurs.

[B15] Miles MB, Huberman AM (1994). Qualitative data analysis: An expanded sourcebook.

[B16] Hanratty B, Hibbert D, Mair F, May C, Ward C, Corcoran G, Capewell S, Litva A (2006). Doctors' understanding of palliative care. Palliat Med.

[B17] Stewart D (1990). Focus groups: Theory and practice.

[B18] Leys M (2003). Health technology assessment: the contribution of qualitative research. Int J Technol Assess Healthcare.

[B19] Hanratty B (2000). Palliative care provided by GPs: the carer's viewpoint. Br J Gen Pract.

[B20] Higginson I (1999). Palliative care services in the community: what do family doctors want?. J Palliat Care.

[B21] Meijler WJ, Van Heest F, Otter R, Sleijfer DT (2005). Educational needs of general practitioners in palliative care: outcome of a focus group study. J Cancer Educ.

[B22] Groot MM, Vernooij-Dassen MJ, Crul BJ, Grol RP (2005). General practitioners (GPs) and palliative care: perceived tasks and barriers in daily practice. Palliat Med.

[B23] O'Connor M, Lee-Steere R (2006). General practitioners' attitudes to palliative care: A Western Australian rural perspective. J Palliat Med.

[B24] Hearn J, Higginson IJ (1998). Do specialist palliative care teams improve outcome for cancer patients? A systematic literature review. Palliat Med.

[B25] Brumley R, Enguidanos S, Cherin DA (2003). Effectiveness of home-based care program for end of life. J Palliat Med.

[B26] Brumley R, Enguidanos S, Jamison P, Seitz R, Morgenstern N, Saito S, McIlwane J, Hillary K, Gonzalez J (2007). Increased satisfaction with care and lower costs: results of a randomized trial of in-home palliative care. J Am Geriatr Soc.

[B27] Enguidanos SM, Cherin D, Brumley R (2005). Home-based palliative care study: site of death, and costs of medical care for patients with congestive heart failure, chronic obstructive pulmonary disease, and cancer. J Soc Work End Life Palliat Care.

[B28] Hibbeler B (2007). Ärztliche Versorgung in Pflegeheimen: Von Kooperationen profitieren alle. Dtsch Arztebl.

[B29] Norberg A, Gillon R (1994). Ethics in the Care of the elderly with dementia. Principles of health care ethics.

[B30] Crooks VA, Agarwal G (2008). What are the roles involved in establishing and maintaining informational continuity of care within family practice? A systematic review. BMC Fam Pract.

