# Supplementary material for: Palliative care for older people – exploring the views of doctors and nurses from different fields in Germany
Source: BMC Palliat Care. 2009 Jun 23;8:7. doi: 10.1186/1472-684X-8-7 (PMC2706814; doi:10.1186/1472-684X-8-7)
Supplement: Additional file 1 — Inclusion and characteristics of the participants. The data provided show the inclusion criteria and the demographics of the participants for each professional group. [file 1472-684X-8-7-S1.doc]

**Table 1: Inclusion and characteristics of the participants**

| **Professions** | **Inclusion criterions** | **Participants** | |
| --- | --- | --- | --- |
|  |  | **code/gender** | **characteristics** |
| general practitioners | Family doctors without additional training in geriatrics or palliative care^1^  Recruitment: teaching practices of Hannover Medical School, State Medical Chamber of Lower Saxony | Ha1-1 / m | 15 years in hospital, family doctor since 13 years |
|  |  | Ha1-2 / m | family doctor since 10 years |
|  |  | Ha1-3 / m | family doctor since 20 years, working in a rural area |
|  |  | Ha2-1 / f | family doctor since 9 years |
|  |  | Ha2-2 / m | family doctor since 7 years |
|  |  | Ha2-3 / f | family doctor since 4 years |
|  |  | Ha3-1 / f | - no information |
| geriatricians | Doctors with specialist training in geriatrics (and/or working fulltime as geriatricians for at least two years)^2^  Recruitment: State Medical Chamber of Lower Saxony, Hospitals for geriatrics in Hanover and Hanover region | Ga1-1 / f | - no information |
|  |  | Ga1-2 / m | training in intensive care medicine and cardiology, since 4 years geriatrician |
|  |  | Ga1-3 / m | for 17 years orthopaedics , since 2 years in geriatrics |
| palliative care physicians | Doctors with specialist training in palliative medicine (and/or with focus on palliative care for at least half of their total working time)^3^  Recruitment: State Medical Chamber of Lower Saxony, palliative care teams, hospices und palliative care units in Hanover and surrounding area | Pa1-1 / f | anesthetist, since 4 years palliative care physician in a university hospital |
|  |  | Pa1-2 / f | GP for 20 years, since 7 years specialist with focus on palliative medicine |
|  |  | Pa1-3 / m | since 34 years anesthetist, since 8 years working with focus on palliative medicine |
| palliative care nurses | Nurses with additional specialist training in palliative care and focus on palliative care with at least half of their working time  Recruitment: palliative care teams, hospices und palliative care units in Hanover and surrounding | Pc1-1 / f | since 26 years nurse |
|  |  | Pc1-2 / f | nurse, since 25 years working in nursing home |
|  |  | Pc1-3 / f | since 11 years working in a hospice |
|  |  | Pc1-4 / f | since 20 years nurse, since 2 years working in a hospice |
|  |  | Pc1-5 / f | for 2 years working in a teaching hospital, since 8 years home care |
|  |  | Pc2-1 / f | since 25 years nurse, since 2 years working in a nursing home |
|  |  | Pc3-1 / m | since 2 years home care nurse |
| general nurses | Nurses without additional training in palliative care and / or geriatrics  Recruitment: in home care teams and nursing homes in Hanover | Pf1-1 / f | since 15 years nurse, since 2 years working in home care |
|  |  | Pf1-2 / f | since 20 years nurse, home care |
|  |  | Pf1-3 / f | since 32 years nurse, since 11 years home care nurse |
|  |  | Pf1-4 / f | for 9 years home care, for 1 ½ years working in a nursing home |
|  |  | Pf1-5 / f | since 5 years home care nurse |
|  |  | Pf1-6 / f | since 20 years nurse, since 2 years working in home care |
|  |  | Pf1-7 / f | since 2 years working in transcultural home care |
|  |  | Pf1-8 / f | since 4 years working in transcultural home care |
|  |  | Pf1-9 / m | since 12 years working in transcultural home care |

According to the regulations of education (Weiterbildungsordnung, WBO) of the Lower Saxony Medical Association.

^2^ Because there are relatively few doctors with formal specialist training in geriatrics (according to WBO) we also included doctors working fulltime for at least two years in specialist geriatric services.

^3^ In Germany, relatively few doctors with specialist training in palliative medicine exclusively work in palliative medicine; most of them work in their primary speciality (e.g. anaesthesiology, oncology, family medicine). So, we included doctors with focus on palliative care for at least half of their total working time.
