# Supplementary material for: Palliative care for older people – exploring the views of doctors and nurses from different fields in Germany
Source: BMC Palliat Care. 2009 Jun 23;8:7. doi: 10.1186/1472-684X-8-7 (PMC2706814; doi:10.1186/1472-684X-8-7)
Supplement: Additional file 2 — Focus groups. The table presents the characteristics of the five monoprofessional and two mixed focus groups. [file 1472-684X-8-7-S2.doc]

**Table 2: Focus groups**

| Focus groups 1-5 **(monoprofessional)** |  | |  | **Focus group 6**  **(mixed)**  total N=12 | **Focus group 7**  **(mixed)**  total N=11 |
| --- | --- | --- | --- | --- | --- |
| group 1:general practitioners (Ha) | participants (n) | | 7 | 3 | 2 |
|  | gender | female | 3 | 2 | 1 |
|  |  | male | 4 | 1 | 1 |
|  | age (average, range) | | 46 years / 36:58 | 47 years / 36:58 | 53 years / 47:58 |
|  | working place | Hanover | 6 | 2 | 1 |
|  |  | Greater Hanover | 1 | 1 | 1 |
| group 2:geriatricians (Ga) | participants (n) | | 3 | 3 | 3 |
|  | gender | female | 1 | 1 | 1 |
|  |  | male | 2 | 2 | 2 |
|  | age (average, range) | | 46 years / 44:48 | 46 years / 44:48 | 46 years / 44:48 |
|  | working place | Hanover | 0 | 0 | 0 |
|  |  | Greater Hanover | 3 | 3 | 3 |
| group 3:palliative care physicians (Pa) | Participants (n) | | 3 | 1 | 2 |
|  | gender | female | 2 | 1 | 1 |
|  |  | male | 1 | 0 | 1 |
|  | age (average, range) | | 53 years / 39:67 | 39 years / 39:39 | 53 years / 39:67 |
|  | working place | Hanover | 2 | 1 | 1 |
|  |  | Greater Hanover | 1 | 0 | 1 |
| group 4:general nurses (Pf) | participants (n) | | 9 | 3 | 3 |
|  | gender | female | 8 | 3 | 3 |
|  |  | male | 1 | 0 | 0 |
|  | age (average, range) | | 37 years / 26:49 | 35 years / 29:45 | 35 years / 29:45 |
|  | working place | Hanover | 9 | 3 | 3 |
|  |  | Greater Hanover | 0 | 0 | 0 |
| group 5:palliative care nurses (Pc) | participants (n) | | 7 | 2 | 1 |
|  | gender | female | 6 | 2 | 1 |
|  |  | male | 1 | 0 | 0 |
|  | age (average, range) | | 42 years / 27:52 | 44 years / 40:48 | 48 years / 48:48 |
|  | working place | Hanover | 6 | 2 | 1 |
|  |  | Greater Hanover | 1 | 0 | 0 |
