# Supplementary material for: Palliative care for older people – exploring the views of doctors and nurses from different fields in Germany
Source: BMC Palliat Care. 2009 Jun 23;8:7. doi: 10.1186/1472-684X-8-7 (PMC2706814; doi:10.1186/1472-684X-8-7)
Supplement: Additional file 3 — Central questions for the focus group discussions. The central questions that were posed during the focus group discussions are described. [file 1472-684X-8-7-S3.doc]

**Table 3: Central questions for the focus group discussions**

| **groups no 1-5** |
| --- |
| Please think of the term “geriatric palliative care” – what do you associate with this term? |
| If you think of your practical experience, which health care professions are involved in palliative care for older people? |
| Please consider the three medical fields palliative care, geriatrics and general practice: What similarities and differences do you see? |
| If you think of your everyday work: How does the cooperation between the parties work and how could the cooperation be improved? |
| What knowledge and skills are needed by the professionals in the field of geriatric palliative care? |
| If you think of the German health system: Where do you see supporting and inhibiting factors for promoting the implementation of geriatric palliative care? |
| How can barriers and obstacles regarding the delivery of palliative care for older people be overcome, and who can contribute? |
| **group no 6** |
| What practical approaches do you see in palliative care for patients with dementia? |
| We learnt from the previous group discussions that for patients with cognitive impairments, decisions on diagnostics and treatment are particularly difficult. What possibilities do you see to improve the decision making process? |
| In some other countries specialist nursing home physicians are established. How do you see the function of these physicians? What are the advantages or disadvantages? |
| Case management is an approach to optimize health care for older chronically ill and multimorbid patients. To your mind, what are components of optimal case management, and who should be responsible? |
| In the previous focus groups the excessive bureaucracy within the German health system was heavily criticized. What suggestions do you have in order to reduce it? |
| **group no 7** |
| Please think about younger and older palliative care patients, e.g. regarding possibilities and limits of diagnostic and treatment approaches. Could you please think about age specific differences? |
| If you think about older male and female patients within the palliative care setting – what are your opinions regarding gender specific differences? |
| What legal problems do you see concerning palliative care for older people? |
| What are your opinions regarding the recently introduced legal right to specialist out-patient palliative care in Germany – to your mind, will it improve the provision of palliative care for the specific group of older patients? |
