# Supplementary material for: Palliative care for older people – exploring the views of doctors and nurses from different fields in Germany
Source: BMC Palliat Care. 2009 Jun 23;8:7. doi: 10.1186/1472-684X-8-7 (PMC2706814; doi:10.1186/1472-684X-8-7)
Supplement: Additional file 4 — Main categories and subcategories. This table shows the four main categories and the subcategories that resulted from the content analysis of the focus group transcripts. [file 1472-684X-8-7-S4.doc]

**Table 4: Main categories and subcategories**

I Stakeholders involved in geriatric palliative care

- - Roles of different medical disciplines
  - Knowledge, skills and expertise

II Target groups

- - Differences between older and younger palliative care patients
  - Gender-related differences

III Inhibiting factors for the realization of geriatric palliative care

- - Health care system-related barriers
  - Bureaucracy

IV Improvement of palliative care for older people

- - More money, time, and communication
  - Nursing home physicians
